# Radiation exposure doses to the surgical team during hip surgery is significantly higher during lateral imaging than posteroanterior imaging: a cadaveric simulation study

**DOI:** 10.1186/s12995-023-00396-0

**Published:** 2023-11-30

**Authors:** Yasuaki Tamaki, Kazuta Yamashita, Daiki Nakajima, Yasuyuki Omichi, Yoshinori Takahashi, Michihiro Takai, Shunsuke Tamaki, Tomohiro Goto, Hiroaki Hayashi, Kosaku Higashino, Yoshihiro Tsuruo, Koichi Sairyo

**Affiliations:** 1 Department of Orthopedics, Institute of Biomedical Sciences, Tokushima University Graduate School, 3-18-15 Kuramoto, Tokushima, 770-8503 Japan; 2 Department of Orthopedics, Tokushima Municipal Hospital, 2-34 Kitajosanjima, Tokushima, 770-0812 Japan; 3 Department of Pharmaceutical and Health Sciences, Kanazawa University Graduate School, Kakuma-Machi, Kanazawa City, Ishikawa 920-1192 Japan; 4 Department of Anatomy, Tokushima University Graduate School, 3-18-15 Kuramoto, Tokushima, 770-8503 Japan

**Keywords:** Radiation, Occupational radiation exposure, Fluoroscopy, Orthopedic surgery, Hip

## Abstract

**Background:**

Fluoroscopy is indispensable when determining appropriate and effective interventions in orthopedic surgery. On the other hand, there is growing concern about the health hazards of occupational radiation exposure. The aim of this cadaveric simulation study was to measure radiation exposure doses to the surgical team during hip surgery.

**Methods:**

We reproduced the intraoperative setting of hip surgery using 7 fresh frozen cadavers (5 male, 2 female) to simulate patients and mannequins to simulate the surgeon, scrub nurse, and anesthesiologist. Six real-time dosimeters were mounted at sites corresponding to the optic lens, thyroid gland, chest, gonads, foot, and hand on each mannequin. The radiation exposure dose to each team member was measured during posteroanterior and lateral fluoroscopic imaging.

**Results:**

Radiation exposure doses to the surgeon were significantly higher during 3 min of lateral imaging than during 3 min of posteroanterior imaging at the optic lens (8.1 times higher), thyroid gland (10.3 times), chest (10.8 times), and hand (19.8 times) (*p* = 0.018, *p* = 0.018, *p* = 0.018, and *p* = 0.018, respectively). During lateral imaging, the radiation doses to the nurse were 0.16, 0.12, 0.09, 0.72, and 0.38 times those to the surgeon at the optic lens, thyroid, chest, gonads, and foot, respectively. The radiation dose to the anesthesiologist was zero at all anatomic sites during posteroanterior imaging and very small during lateral imaging.

**Conclusions:**

Radiation exposure dose was significantly higher during lateral imaging up to 19.8 times comparing to the posteroanterior imaging. It is effective to reduce the lateral imaging time for reducing the intraoperative radiation exposure. In addition, appropriate distance from fluoroscopy resulted in very low exposure for nurses and anesthesiologists. Surgeon should pay attention that surgical staff do not get closer than necessary to the irradiation field.

## Introduction

Fluoroscopy is indispensable when determining appropriate and effective interventions in orthopedic surgery. The use of fluoroscopy in hip surgery has expanded to include not only trauma surgery but also arthroscopy, osteotomy, and various other procedures. The surgical team, including surgeons, nurses, and anesthesiologists, are regularly exposed to direct or scatter radiation for extended periods when performing fluoroscopic-guided procedures. There is growing concern about the health hazards of occupational radiation exposure. Compared with other radiation-exposed workers, orthopedic surgeons are at significantly higher risk of developing malignant disease [1]. Moreover, there have been several reports on the association between occupational radiation exposure and various adverse events, including cataracts, thyroid cancer, and skin cancer [2–5]. However, orthopedic surgeons generally have low awareness of the need to protect themselves from radiation exposure [6]. In a survey by Saroki et al., most surgeons gave incorrect answers to questions about which C-arm positions and settings result in the lowest radiation doses to the surgeon and patient and 91.2% indicated that orthopedic surgeons need to be more informed about radiation safety [7]. These previous studies show that it is necessary to raise awareness of radiation exposure among orthopedic surgeons based on precise knowledge and to make efforts to minimize exposure during surgery, not only for the health of surgeons themselves but for that of the whole surgical team.

It is well known that exposure time, distance from the source, and barriers to exposure are important factors for reducing the amount of radiation exposure [8]. Therefore, it can be anticipated that surgeons, who are closest to the radiation source during surgery, would be exposed to a larger radiation dose than nurses and anesthesiologists, who work further away from the source. However, to our knowledge, there is limited literature on the precise radiation exposure doses to each member of the surgical team. Furthermore, more information is needed on radiation exposure at different anatomic sites, given that radiation sensitivity varies from one site to another.

The purpose of this cadaveric simulation study was to measure radiation exposure doses to the surgeon, nurse, and anesthesiologist at the optic lens, thyroid gland, chest, gonads, foot, and hand during fluoroscopic-guided hip surgery. Furthermore, we also verified the amount of change in exposure dose depending on C-arm position and irradiation time.

## Materials and methods

This research has been approved by the IRB of the authors’ affiliated institutions and performed in accordance with the principles of the Declaration of Helsinki. In line with standard practice, written informed consent was obtained from all individuals and their families when they donated their body to our institution for research purposes.

Seven fresh frozen cadavers (5 male, 2 female) were used in the study. Mean age at the time of death was 72.7 (range, 51–94) years. Mean height and weight were 162.9 (range, 147–175) cm and 58.7 (range, 40–100) kg, respectively. Mean anteroposterior diameter of the hip was 14.7 (range, 12–18) cm and mean mediolateral diameter was 22.9 (range, 15–30) cm. We accurately reproduced the working environment of hip surgery using these cadaveric specimens.

**Fig. 1:**
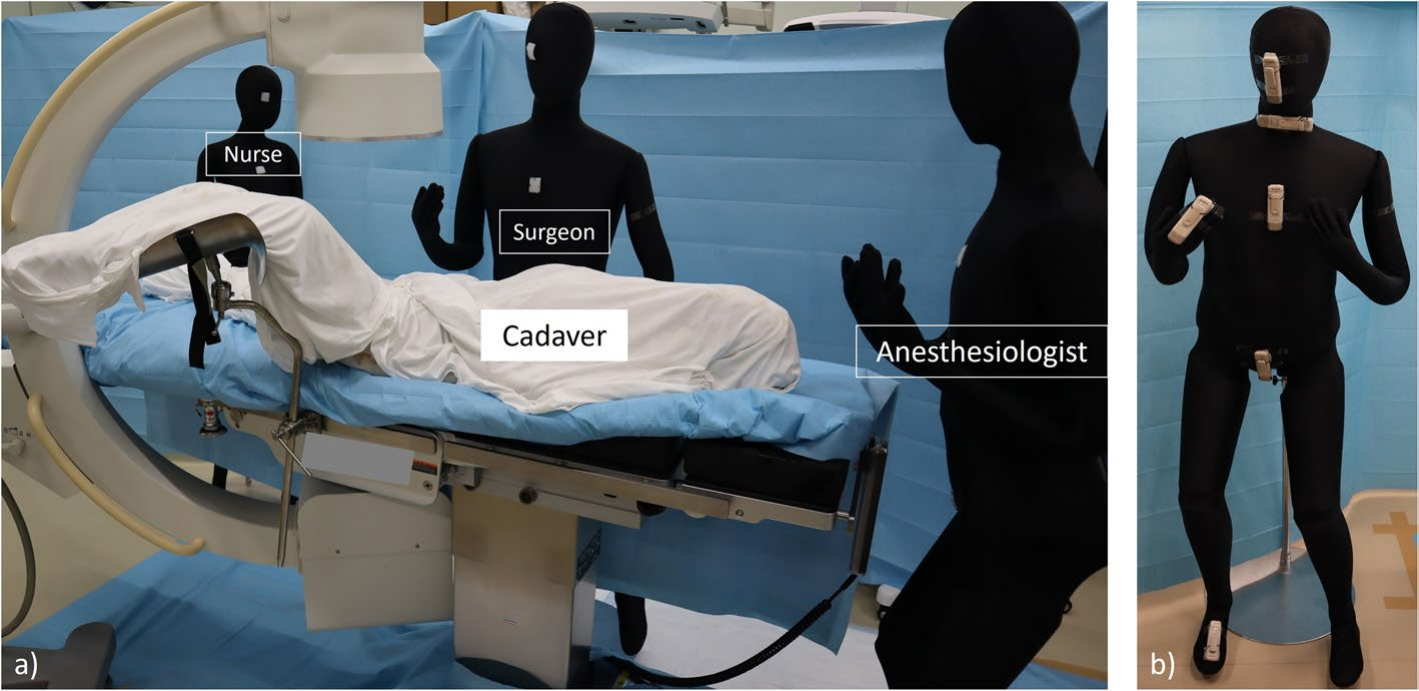
**a** This photo was shown the position of the C-arm fluoroscope and mannequins simulating the surgeon, nurse and anesthesiologist. **b** Six dosimeters were placed on the mannequin simulating the surgeon, nurse and anesthesiologist

**Fig. 2:**
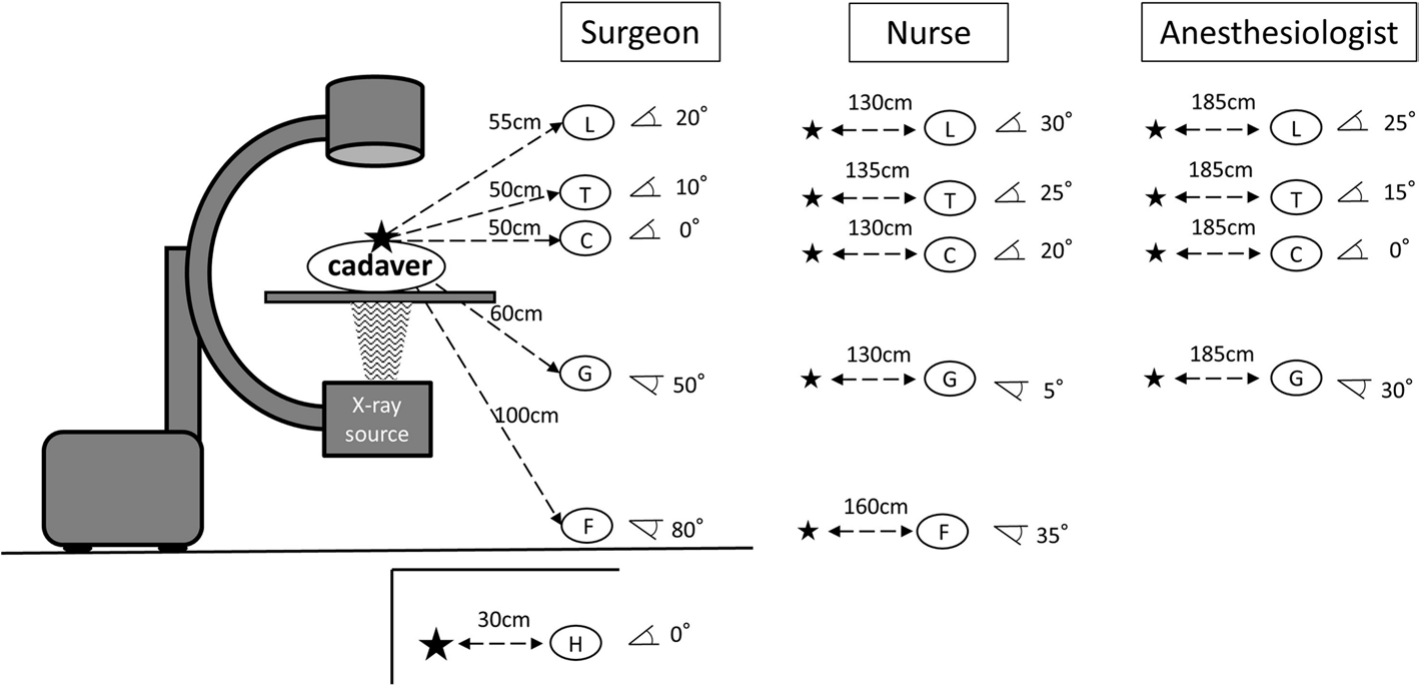
Position of the fluoroscope and dosimeters during posteroanterior imaging. The distance and angle between the center of the irradiation field and each dosimeter was shown. L: lens of the eye; T: thyroid; C: chest; G: gonad; F: foot; H: hand

**Fig. 3:**
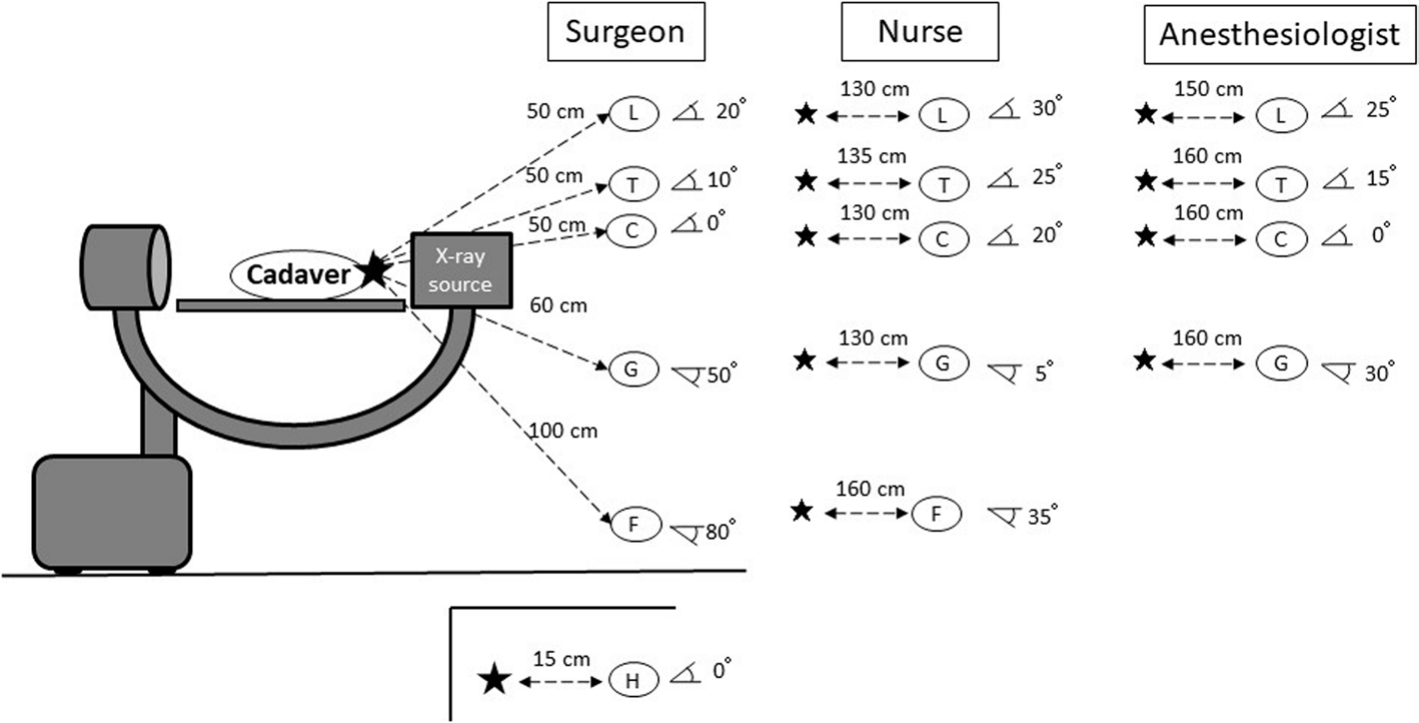
Position of the fluoroscope and dosimeters during lateral imaging. The distance and angle between the center of the irradiation field and each dosimeter was shown. L: lens of the eye; T: thyroid; C: chest; G: gonad; F: foot; H: hand

Mannequins were used to simulate the surgeon, scrub nurse, and anesthesiologist and were placed in each working position (Fig. 1a). Six real-time dosimeters (MYDOSE mini, Hitachi Aloka Medical, Tokyo; detectable range, 1 µSv to 9999 mSv) in one setting were mounted at sites corresponding to the optic lens, thyroid gland, chest, gonads, foot, and hand on each mannequin (Fig. 1b). The hand dosimeter was omitted for the nurse and anesthesiologist because their hand positions are not constant during surgery. The position of each dosimeter was defined by the distance and angle from the center of the irradiation field in order to maintain a constant position in all settings (Figs. 2 and 3).

An adjustable radiolucent surgical table (MOT-5602BW, Mizuho Medical Co. Ltd., Tokyo, Japan) was used to position the cadavers. All radiation exposures to the cadavers were performed using C-arm fluoroscopy (BV Vectra, Philips, Eindhoven, Netherlands). The C-arm fluoroscopic system was set to automatic mode; all technical factors, including kilovolt (kV) peak and milliampere (mA) values, were automatically adjusted to optimize image quality.

Radiation exposure doses were measured during posteroanterior (PA) and lateral fluoroscopic imaging (Figs. 2 and 3). During PA imaging, the X-ray source was placed under the table. During lateral imaging, the mannequins simulating the surgeon and nurse were placed to the side of the X-ray source. The cadaver was irradiated for 3 min from each source position with the beam centered on the femoral head. The irradiation time was determined by referring to past reports (2.20–3.89 min per surgery for femoral intertrochanteric fracture) and assuming the intraoperative usage time [9, 10]. The radiation exposure dose to the surgeon during 1 min was also measured to establish the relationship between irradiation time and exposure dose.

Differences in radiation exposure dose between PA imaging and lateral imaging were examined for statistical significance using the Wilcoxon signed rank test. All statistical analyses were performed using SPSS for Windows version 24.0 (IBM Corp., Armonk, NY, USA). A *p*-value < 0.05 was considered statistically significant.

## Results

We measured the radiation exposure doses to the surgeon, nurse, and anesthesiologist during PA and lateral imaging. Mean tube voltages during PA and lateral imaging were 61.1 (range, 57–73) kV and 69.7 (range, 59–96) kV, respectively. Mean electrical currents during PA and lateral imaging were 1.4 (range, 1.02–2.76) mA and 2.1 (range, 1.20–2.97) mA, respectively.

### Radiation exposure doses to the surgeon

The radiation exposure doses to the surgeon’s optic lens, thyroid gland, chest, gonads, foot, and hand are summarized in Table 1. The radiation exposure dose at each anatomic site was on average 3.05 (range, 2.88–3.37) times greater for 3 min of exposure than for 1 min. The radiation dose tended to be approximately proportional to the irradiation time. The radiation exposure dose to the optic lens, thyroid gland, chest, and hand during 3 min of exposure was significantly greater during lateral imaging than during PA imaging (*p* = 0.018, *p* = 0.018, *p* = 0.018, and *p* = 0.018, respectively). For 3 min of exposure, the respective radiation doses to the optic lens, thyroid gland, chest, and hand were 8.1, 10.3, 10.8, and 19.8 times higher during lateral imaging than during PA imaging. The radiation exposure dose to the gonads was 2.7 times higher during lateral imaging than during PA imaging; however, the difference was not statistically significant (*p* = 0.051). The radiation dose to the foot was similar between PA and lateral imaging (*p* = 0.933).

**Table 1:** Radiation exposure doses to the surgeon during 1 and 3 min of posteroanterior and lateral fluoroscopic imaging

	Posteroanterior (µSv)		Lateral (µSv)		
	1 min	3 min	1 min	3 min	*p*-value
Lens	3.4 ± 1.6 (2–7)	10.4 ± 4.6 (6–21)	28.0 ± 33.7 (5–106)	84.3 ± 100.9 (15–318)	0.018
Thyroid	3.7 ± 4.0 (0–13)	11.3 ± 11.9 (2–39)	38.7 ± 43.1 (5–138)	116.3 ± 129.2 (16–414)	0.018
Chest	4.9 ± 3.9 (2–14)	15.1 ± 11.5 (6–42)	54.6 ± 59.4 (5–187)	163.4 ± 1 78.5 (16–561)	0.018
Gonads	1.6 ± 1.6 (0–5)	5.4 ± 4.5 (1–15)	5.0 ± 5 .9 (1–19)	14.4 ± 17.7 (3–57)	0.051
Foot	2.3 ± 2.9 (0–9)	7.0 ± 8.5 (1–27)	2.6 ± 2.2 (0–6)	7.7 ± 6.1 (0–18)	0.933
Hand	15.1 ± 8.3 (4–30)	45.6 ± 24.6 (12–90)	301.0 ± 445.3 (26–1380)	902.7 ± 1336.1 (76–4140)	0.018

All data are shown as the mean ± standard deviation (range). *P*-values indicate the statistical significance of differences in radiation dose after 3 min of posteroanterior imaging and lateral imaging

### Radiation exposure doses to the scrub nurse

The radiation exposure doses to the scrub nurse’s optic lens, thyroid gland, chest, gonads, and foot are summarized in Table 2. The respective radiation doses to the optic lens, thyroid gland, and chest were 8.1, 10.8, and 6.8 times higher during lateral imaging than during PA imaging; the differences were statistically significant (*p* = 0.018, *p* = 0.018, and *p* = 0.043). There was no statistically significant difference in radiation exposure dose to the foot between PA and lateral imaging (*p* = 0.157). After 3 min of exposure during lateral imaging, the respective radiation exposure doses to the nurse were 0.16, 0.12, 0.09, 0.72, and 0.38 times those to the surgeon at the optic lens, thyroid gland, chest, gonads and foot.

**Table 2:** Radiation exposure doses to the nurse during 3 min of posteroanterior and lateral fluoroscopic imaging

	Posteroanterior (µSv)	Lateral (µSv)	*p*-value
Lens	1.7 ± 2.0 (0–6)	13.7 ± 12.8 (3–42)	0.018
Thyroid	1.3 ± 2.0 (0–6)	14.1 ± 15.4 (2–48)	0.018
Chest	2.1 ± 2.0 (0–6)	14.3 ± 15.3 (2–48)	0.043
Gonads	1.7 ± 2.2 (0–6)	10.4 ± 13.6 (1–42)	0.075
Foot	2.0 ± 3.0 (0–9)	2.9 ± 3.3 (0–9)	0.157

All data are shown as the mean ± standard deviation (range)

### Radiation exposure doses to the anesthesiologist

The radiation exposure doses to the anesthesiologist’s optic lens, thyroid gland, chest, gonads, and foot are summarized in Table 3. There was no radiation exposure at any anatomic site during PA imaging and only a very small amount during lateral imaging with no statistically significant difference in radiation dose according to whether the imaging was PA or lateral.

**Table 3:** Radiation exposure doses to the anesthesiologist during 3 min of posteroanterior and lateral fluoroscopic imaging

	Posteroanterior (µSv)	Lateral (µSv)	*p*-value
Lens	0	1.9 ± 2.6 (0–6)	0.102
Thyroid	0	1.0 ± 1.3 (0–3)	0.102
Chest	0	0.9 ± 1.4 (0–3)	0.157
Gonads	0	0.4 ± 1.0 (0–3)	0.317
Foot	0	0	-

All data are shown as the mean ± standard deviation (range)

## Discussion

In this simulation study, we measured the radiation exposure dose to the surgeon, nurse, and anesthesiologist in the setting of fluoroscopic hip surgery using cadaveric specimens. The surgeon was exposed to the most of radiation (up to 11.4 times more than to the nurse). For all surgical team members, the exposure dose was up to 19.8-fold higher during lateral imaging than during PA imaging.

According to the recommendations of the International Commission on Radiological Protection, the annual occupational radiation exposure thresholds are 20 mSv (100 mSv/5 years, maximum 50 mSv/year) for the optic lens, 500 mSv for the thyroid gland, and 500 mSv for the hand. Surgeons should be aware of the stochastic effects of radiation, that is, the long-term risk of cancer and genetic defects associated with repeated exposure to ionizing radiation [11]. The probability of stochastic effects of radiation exposure increases with radiation dose. Therefore, the exposure dose received during fluoroscopy should be minimized in accordance with the ALARA (as low as reasonably achievable) principle [12, 13].

It is well known that distance from the radiation source is an important factor in radiation exposure dose [8]. The simplest way of minimizing radiation exposure is to stay as far away from the radiation source as possible. Alonso et al. reported that outside a 2-m radius from the radiation source, there is little or no risk of radiation exposure [14]. Mehlman et al. reported that unprotected individuals working 70 cm (24 inches) or less from a fluoroscopic beam received significant amounts of radiation [15]. Moreover, they found that personnel working 90 cm away from the beam were exposed to a low radiation dose and that those working 150 cm away received almost no radiation. In the present study, a surgeon working 50 cm from the X-ray source received the highest radiation dose; specifically, the dose to the surgeon was up to 11.4 times higher than that to a nurse working 130 cm away. Although a scrub nurse working 130 cm from the source was exposed to a small amount of radiation, an anesthesiologist working 185 cm from the source received almost none. This finding is consistent with previous reports and suggests that the nurse and anesthesiologist can limit their risk of stochastic effects of radiation to maintain an appropriate distance from radiation source during surgery.

Surgeons should make every effort to reduce their radiation exposure, given their need to work close to the radiation source. In our study, the highest radiation doses were recorded at the surgeon’s hand. Although the dose of scatter radiation to the hand in our study was small comparing to annual occupational radiation exposure thresholds according to the recommendations of the International Commission on Radiological Protection (500 mSv), we only evaluated the scatter radiation not included the direct radiation. It is common for surgeon to be expose to direct radiation, such as in the process of repairing fracture site. Thus, we should consider intraoperative radiation exposure including the effects of direct radiation. When the surgeon’s hand strays into the irradiation field, the direct radiation dose is 20–100 times greater than the scatter dose [16–19]. Therefore, surgeons should keep their hands as far away from the irradiation field as possible during surgery.

Another important finding in this study was that the radiation dose was significantly higher during lateral imaging than during PA imaging, which can be explained by the greater distance across which the X-ray beam needs to travel in the lateral direction. The mean PA diameter of the hip was 14.7 cm and the mean mediolateral diameter was 22.9 cm in the present study. Furthermore, the voltage and amplitude values for the C-arm were higher during lateral imaging than during PA imaging. Therefore, greater caution regarding radiation exposure is needed during lateral imaging.

This study has several limitations. First, the cadavers used were relatively small (mean height, 162.9 cm; mean weight, 58.7 kg). The irradiation dose increases with increasing body size. Therefore, physique should be considered when interpreting the results of this study. Second, the shielding effect of radiation equipment, such as gloves and goggles, was not considered. There are reports showing that personal protective equipment reduces an individual’s exposure to radiation [20, 21]. Therefore, occupational radiation exposure dose should be considered in light of whether protective equipment is worn.

In conclusion, this study has quantified the scatter radiation exposure doses to the surgical team at several anatomic sites during hip surgery. Because surgeons are exposed to a larger amount of radiation than other members of the surgical team, they should implement measures to reduce their exposure and the risk of stochastic effects of radiation. Based on our results, reducing the duration of the lateral imaging may be one of the effective methods for reducing intraoperative radiation exposure dose. In addition, the nurse and anesthesiologist can avoid health hazards from occupational radiation exposure by maintaining an appropriate distance from the radiation source.

## Data Availability

The data that support the findings of this study are available from the corresponding author upon reasonable request.

## Abbreviations

PA: Posteroanterior
ALARA: As low as reasonably achievable
